# Gene expression subtraction of non-cancerous lung from smokers and non-smokers with adenocarcinoma, as a predictor for smokers developing lung cancer

**DOI:** 10.1186/1756-9966-27-45

**Published:** 2008-09-24

**Authors:** David Stav, Ilan Bar, Judith Sandbank

**Affiliations:** 1 Pulmonary Institute, Assaf Harofeh Medical Center, Zerrifin 70300, Israel; 2 Deparment of Chest Surgery, Assaf Harofeh Medical Center, Zerrifin 70300, Israel; 3 Deparment of Pathology, Assaf Harofeh Medical Center, Zerrifin 70300, Israel

## Abstract

**Background:**

Lung cancer is the commonest cause of cancer death in developed countries. Adenocarcinoma is becoming the most common form of lung cancer. Cigarette smoking is the main risk factor for lung cancer. Long-term cigarettes smoking may be characterized by genetic alteration and diffuse injury of the airways surface, named field cancerization, while cancer in non-smokers is usually clonally derived. Detecting specific genes expression changes in non-cancerous lung in smokers with adenocarcinoma may give us instrument for predicting smokers who are going to develop this malignancy.

**Objectives:**

We described the gene expression in non-cancerous lungs from 21 smoker patients with lung adenocarcinoma and compare it to gene expression in non-cancerous lung tissue from 10 non-smokers with primary lung adenocarcinoma.

**Methods:**

Total RNA was isolated from peripheral non-cancerous lung tissue. The cDNA was hybridized to the U133A GeneChip array. Hierarchical clustering analysis on genes obtained from smokers and non-smokers, after subtracting were exported to the Ingenuity Pathway Analysis software for further analysis.

**Results:**

The genes subtraction resulted in disclosure of 36 genes with high score. They were subsequently mapped and sorted based on location, cellular components, and biochemical activity. The gene functional analysis disclosed 20 genes, which are involved in cancer process (*P *= 7.05E-5 to 2.92E-2).

**Conclusion:**

Detected genes may serve as a predictor for smokers who may be at high risk of developing lung cancer. In addition, since these genes originating from non-cancerous lung, which is the major area of the lungs, a sample from an induced sputum may represent it.

## Background

Lung cancer is the commonest cause of cancer death in developed countries and throughout the world. Worldwide, the estimated number of new lung cancer cases in 2002 is 1.2 million (12.3% of all new cancer cases). Over 90% of these new cases will die as a result of the disease [1]. The death rate for lung cancer exceeds the combined total for breast, prostate and colon cancer in developed countries [1]. Cigarette smoking is the main risk factor for lung cancer, accounting for about 90% of cases in men and 70–85% of cases in women [2]. Genetic risk factors contribute to an individual's susceptibility to lung cancer, which is illustrated by the fact that about 16% of long-term smokers will develop lung cancer [3]. Unfortunately, no effective clinical tests are available for early detection of lung cancer. Smoking cessation programs are critical, but ex-smokers continue to have a higher risk to develop lung cancer. Even more than 40 years after cessation compared with never-smokers [4]. These days ex-smokers comprise almost 50% of all new lung cancer cases in developed countries, indicating a strong need for a search for new means of early diagnosis in lung cancer and chemoprevention in this high-risk group (4). Nevertheless, an important issue for these approaches is the appropriate selection of an optimal high-risk population.

Long-term carcinogenic exposure (cigarettes smoke) is characterized by genetic alteration and diffuse injury of the airways surface [5]. These changes in epithelium can give rise to cancer at multiple points, for that reason named field cancerization. Genetic changes detected in premalignant lesions or malignant in one region of the field, translate into an increased risk of cancer development throughout the entire field. Therefore, it can be concluded that cigarettes smoking may induce field cancerization whereas cancer in non-smokers is clonally derived, i.e. by single cell transformed is the ancestor of all cells that compose the neoplasm [6-8].

The consequence of this is that the non-cancerous tissue in non-smoker patient with primary lung cancer should not show any alteration compared to normal, whereas in smokers due to field cancerization morphological and genetic alteration may possibly be observed. Probably, this genetic alteration in smokers could serve for early detection of lung cancer.

Identification of individuals at greatest risk of developing lung cancer could enhance the efficacy of intervention modalities, thereby greatly reducing mortality from this disease. One strategy for identifying these people is to establish molecular markers that reflect the severity of their cancerization field.

Microarray can simultaneously measure the expression of thousands of mRNAs. They are used in many biological fields and in different species. This high-throughput technique can be used to predict the function of unknown genes, in medical diagnostics, in biomarker discovery, to infer networks from the regulatory interactions between genes, and to investigate the mechanisms by which a drug, disease, mutation and environmental condition affects gene expression and cell function. Large datasets are produced, particularly from whole-genome arrays, and public databases hold substantial quantities of gene-expression information [9,10].

Adenocarcinoma (AC) is becoming the most common form of lung cancer. It has increased relative to other histological types of lung cancer over the last several decades [11-14].

These observations lead us to investigate the gene expression in non-involved lungs from patients with adenocarcinoma and compare it to gene expression in non-involved lung tissue from non-smokers. We used the U133A GeneChip array (Affymetrix, Santa Clara, CA), GeneSpring analysis software and the Ingenuity Pathway Analysis software.

## Methods

### Patient Lung Tissue Samples

We obtained lungs tissue from 32 patients with adenocarcinoma of lung who underwent pulmonary lobectomy (Table 1). All were pre-operatively diagnosed as having lung adenocarcinoma by bronchoscopy or fine needle aspiration. Tumors were carefully staged preoperatively, and clinically believed to be N2 node negative by, computerized tomography, positron emission tomography, or more frequently mediastinoscopy. None of the patients received preoperative anti-cancer therapy. Surgery included mediastinal lymph node sampling. The histological classification was made according to the standard WHO criteria. The affected lobes were removed; all non-involved lung tissue samples were obtained in accordance with Institutional Review Board guidelines. The tissues were carefully inspected, and declared histologically normal (*never-smokers*) or non-cancerous (*smokers*).

**Table 1:** Profile of Lung adenocarcinoma patients

**Age/gender**	**Smoker**	*FEV1%	**Tumor size (mm)**	**Lymph node**	**Stage**
80/M	Yes	55	25	T 1N 0	I a
76/M	Yes	72	30	T 1N 0	I a
70/F	Yes	48	24	T 1N 0	I a
58/M	Yes	82	22	T 1N 0	I a
62/M	Yes	35	28	T 1N 0	I a
54/M	Yes	45	24	T 1N 0	I a
74/F	Yes	85	32	T 2N 0	I b
60/M	Yes	80	20	T 1N 0	I a
68/F	Yes	88	25	T 1N 0	I a
74/F	Yes	76	30	T 1N 0	I a
65/F	Yes	82	15	T 1N 0	I a
72/M	Yes	88	35	T 2N 0	I b
54/M	Yes	45	40	T 2N 0	I b
62/M	Yes	72	40	T 2N 0	I b
64/M	Yes	62	35	T 2N 0	I b
78/F	Yes	78	45	T 2N 0	I b
45/M	Yes	72	35	T 2N 0	I b
54/M	Yes	46	35	T 2N 0	I b
75/M	Yes	72	35	T 2N 0	I b
73/F	Yes	72	75	T 2N 0	I b
72/M	Yes	56	35	T 2N 1	II b
54/M	No	85	18	T 1N 0	I a
68/F	No	106	15	T 1N 0	I a
68/F	No	106	15	T 1N 0	I a
68/F	No	100	25	T 1N 0	I a
72/M	No	104	37	T 2N 0	I b
78/F	No	112	45	T 2N 0	I b
79/F	No	96	40	T 2N 0	I b
68/M	No	84	35	T 2N 0	I b
85/M	No	90	35	T 2N 0	I b
78/F	No	96	24	T 1N 1	II a

* = % of predicted

FEV_1 _= forced expiratory volume in first second

### RNA isolation and microarray hybridization

RNA isolation and microarray hybridization. Total RNA was isolated from lung cancer tumor tissue and adjacent non cancerous lung tissue using RNeasy midi kit (Qiagen sciences Maryland USA). cDNA was synthesized from 8 ug total RNA using the SuperScript double stranded cDNA synthesis kit (Invitrogen, Carlsbad CA, USA), and T7-oligo(dT)_24 _primer, according to the manufacturer's instructions. Biotynilated cRNA was synthesized using the "ENZO bioarray high yield RNA transcript labeling kit". (Enzo life sciences Inc., Farmingdale NY USA). The fragmented probe was hybridized to the Affymetrix Human genome U133A Genechip, according to the manufacturer's protocol (Affymetrix inc. Santa Clara, CA USA). The microarrays were scanned using a confocal scanner (Affymetrix Inc.). 18S ribosomal RNA was used as an endogenous control. A single weighted mean expression level for each gene was derived by using Microarray Suite 5.0 software (Affymetrix).

### GeneSpring hierarchical clustering analysis

Expression data were normalized using GeneSpring 7.0 software (Silicon Genetics inc. Redwood CA USA). Gene expression data were normalized in 2 steps. (1) Per chip normalization: and (2) Per gene normalization. We compared gene expression originating from normal lung to non-cancerous lung. More than three fold in expression were considered as significant and used for further analysis.

#### RT-PCR

The seven genes (FYN, MYCN, BRCA1, CD44, Cyclin B2, CDK5RAP3 and RAGE), were further analyzed using quantitative RT-PCR. Cancerous and non cancerous lung tissues from eight randomly chosen patients were studied. 1^st ^strand cDNA was synthesized from 2 ug total RNA by random priming, using the Superscript II cDNA synthesis kit (Invitrogen, Carlsbad CA, USA) according to the manufacturer's instructions. RT PCR reactions were carried out using Taq-Man "Assay on demand" gene expression primers and probe sets; results were analysed by GeneAmp 5700 SDS software (Applied Biosystems, Branchburg, NJ USA). The assays used were: FYN- PPH00147A_ml, MYCN- PPH10927A_ml, BRCA1- PPH00322B_ml, CD44- PPH00114A_ml, RAGE – Hs0015395_m1, Cyclin B2 – Hs00270424_m1 and CDK5RAP3 – Hs01003183_g1. 18S ribosomal RNA was used as an endogenous control. (Hs99999901_s1). The RT-PCR results were analyzed using the comparative threshold cycle (*C*_T_) method. The "relative Quantity" (RQ) of each gene was calculated as follows: RQ = $2^{-\Delta \Delta C_{\text{T}}}$. (15)

### Ingenuity Pathway analysis

A network pathway is initiated by the gene with the highest specificity of connections, and is propagated according to the descent of the specificity. Individual significant pathways identified by a statistical likelihood calculation (P < 0.0001) were merged to represent the biological processes. The Ingenuity Pathways Knowledge Base (KB) is the largest curetted database of previously published findings on mammalian biology from the public literature (Ingenuity Systems). Reports on individual studies of genes in human, mouse or rat were first identified from peer-reviewed publications, and findings were then encoded into ontology by content and modeling experts. Manual extraction and curation probably results in more specific and comprehensive interactions, with far fewer false-positives than automated alternatives (for example, natural language processing and high-throughput screening).

Identification of significant pathways in biological processes: The following steps were used: (1) Genes identified as significant from the experimental data sets were overlaid onto the interactome. Focus genes were identified as the subset having direct interaction(s) with other genes in the database. (2) The specificity of connections for each focus gene was calculated by the percentage of its connections to other significant genes. The initiation and growth of pathways proceeded from genes with the highest specificity of connections. Each pathway had a maximum of 35 genes. (3) Pathways of highly interconnected genes were identified by statistical likelihood using the following equation:

$$\text{Score}=-\log_{10}\left(1-\sum_{i=0}^{f-1}\frac{C(G,i)C(N-G,s-i)}{C(N,s)}\right)$$

Where N is the number of genes in the genomic network, of which G are focus genes, for a pathway of s genes, f of which are focus genes. C(n,k) is the binomial coefficient. (4) Pathways with a score greater than 4 (P < 0.0001) were combined to form a composite network representing the underlying biology of the process.

### Network and gene ontology analysis

The genes, which were most differentially expressed between smokers and non – smokers, were used for network and gene ontology analysis. These genes were exported to the Ingenuity Pathway Analysis (IPA) software (Ingenuity Systems, Mountain View, CA). Of the 36 genes, 35 were mapped and sorted based on location, cellular components, and reported or suggested biochemical, biologic, and molecular functions using the software . The identified genes also were mapped to genetic networks available in the Ingenuity database and then ranked by score. The score is the probability that a collection of genes equal to or greater than the number in a network could be achieved by chance alone. A score of 3 indicate that there is 1/1000 chance that the focus genes are in a network due to random chance. Therefore, score of 3 or higher have a 99.9% confidence level of not being generated by random chance alone. It is possible for a pathway to be both up-regulated and down-regulated, perhaps because of a block in the pathway where genes above and below the block respond differently. Visualizing the results on pathways will assist the identification of genes that are missing from a pathway.

## Results

**Figure 1:**
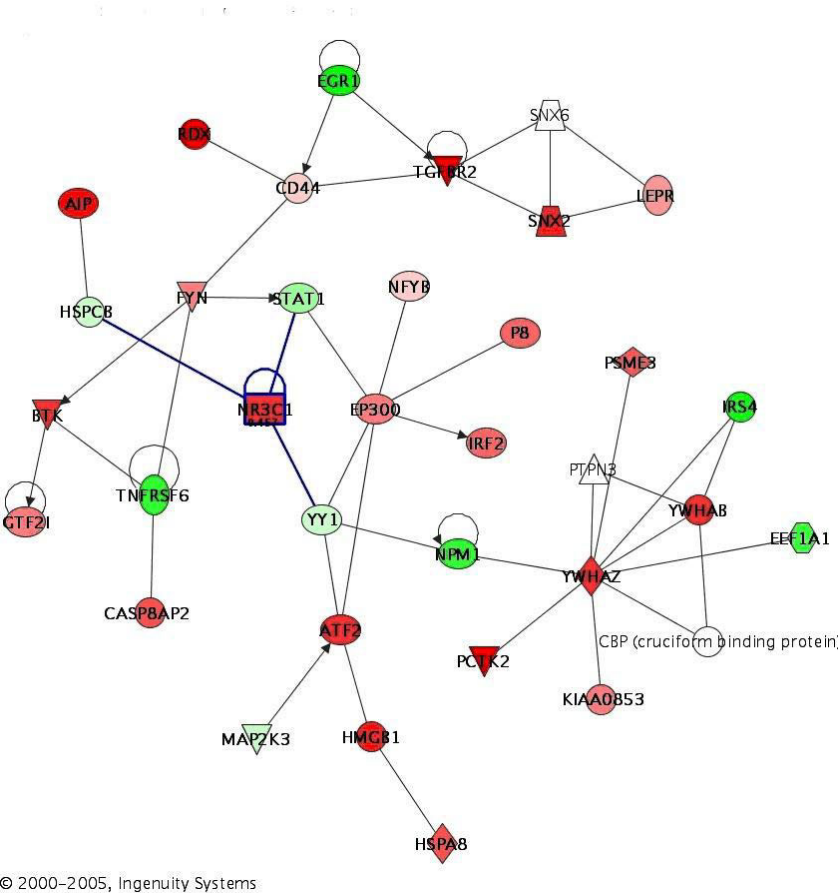
**Gene network is from IPA analysis**. Gene interaction is shown, red colored up-regulated, green colored suppressed. Color intensity is relative to expression. Genes along canonical pathways that were not changed at transcript level are not colored. The arrow pointed toward the direction of positive regulation.

The clinical profile of the patients is shown in Table 1. This includes patients' pulmonary function data, which showed that 12 smokers had chronic obstructive pulmonary disease (COPD)(FEV_1 _< 75%), whereas none of the non-smokers own this limitation. GeneSpring hierarchical clustering was applied to gene expression profiles. We subtracted highly expressed genes of non-smokers from smokers this disclosed 36 genes that were expressed differently between smoker and non-smokers. The Ingenuity analysis and sorting of the 36 genes according theirs expression (bolded and italic over-expressed) or suppressed (normal) while forming networks (Table 2). The first network includes very high scored and focus genes, which are involved in cell death, cancer and inflammatory processes. The following networks with lower score (but still high) and focus genes, included genes that are involved in cellular assembly and organization, cellular function and maintenance. The illustrated network (Fig. 1) (serve as a paradigm) demonstrates the intensity of genes over-expressed (red) or suppressed (green) in smokers compared to non-smokers, and their genes network formation. The network includes genes that are cancer promoters and tumor suppressors such as FYN HSPA8, YWHAZ, LEPR and TGFBR2, IRF2, EP3000 respectively. The suppression of the genes EGR1 and STAT1 correlates with active tumor formation. Many networks include fewer genes in spite of being scored high. This is probably due to lack of knowledge.

**Table 2:** Genes in networks

Network	Genes in Network	Score	Focus Genes	Top function
1	AIP, ATF2, BTK, CASBAP2, CD44, EEF1A1, EGR1, EP300, EPHA2, TF21, HMGB1, HSPA8, HSPCB, IRF2, IRS4, KIAA0853, LEPR, MAP2K3, NFYB, NPM1, NR3C1, P8, PCTK2, PECAM1, PSME3, PTPN11, RDX, SNX2, STAT*, TGFBR2, TNFRSF6, YWHAZ*, YY1	**51**	**35**	Cell death, Cancer, Inflammatory diseases
2	*CCT2*, **CD3Z, CDK2**, *COL6A1, COL6A2*, **COL6A3**, *EIF5*, **EWSR1**, *FCGR3B*, **FGF2**, *FGFBP1*, **FMR1**, FUSIP1, GNA11, GNA13, GNB1, INPP5E, M11S1*, *MAPKAP1*, **Pkci**, *PPR1R12A*, **PRKCZ**, *RAI14, RBM4*, RGS13, RMOQ, SFN*, SKP1A, SQSTM1, **STX4A**, *SYPL, UBE2A*, **VAMP2**, *VAPA*, **YWHAG**	**26**	**31**	Cellular assembly and organization, Cellular function and maintenance, Cell signaling
3	ATF2, C19orf2, DHX9, DR1, RBLN1, FHL1*, **FN1**, **HBA1**, **HBA2**, HBB*, **HBE1, HBG1**, *HBG2*, **HBZ**, *HHEX, HMGB1, HNRPA1, HNRPC*, **IL6**, *LRPPRC*, **MTPN**, *NRP1, PAPOLA*, **POLR2A**, **POLR2F**, *RBPSUH*, **REL, RNU2, RPS25, SMARCB1**, *SNAPC2*, **TBP**, *TCF21*, *TNXB*, **VEGFB**	**18**	**19**	Cell to cell signaling and interaction. Cell morphology.
4	ADAB1*, AK2, ALCAM, AQP1, **CDC2, CDKN2A**, *CLU*, *CDP*, **FOS**, *GD12*, **MIF, MYBL2**, **MYC, MYCN**, *PRDX1*, **PRDX2**, **PRKACB**, *PRKARIB, RBMS1, RPL7A, RPS16, SUMO2, TPM1*, **TXN**, *UNR*	**15**	**15**	Cellular growth and proliferation, cellular and connective tissue development
5	*CCL20, CD44*, **CDC2, CEPBP**, *CXCL1*, **FOS**, *G3BP2*, **ICAM1**, *KIAA0992*, **MCF2, MET**, *MGAT4A*, **MTPN, NFKB1**, **NFKB1B**, *NR3C1, OK/SW-cl.56*, **PSMA7**, *RDX*, **RELA**, *RHOC*, *RPS24, SCAMP1*, **SMARCA4, SNAP23**, STAT1*, **STX4A, STX6, TLR2**, *TNF*, TRA1*, TUBA6, VAMP3*, **VIL2**, *WASF2*	**15**	**17**	Cell death, Immunological disease
6	*ALDH1A1*, **ANXA2**, **BRAF, BRCA1, CCNB1, CDK4**, **CDKN2A**, *DDA3, DHX9*, **HMGA1**, *IDI1*, **IPO7**, *KPNA4*, **KPNB1**, *LMO4*, **MMP2, MYCN, MMI**, *NPM1*, *RASGRP1*, **RBBPS**, *RPL12*, **RPL19**, *RPL23A, RPL3*, *RPL31, RPL37A, RPL39*, **RPL5, RPLPO**, RPS2, SFN*, TBX2*, **TP73, TP73L**	**15**	**17**	Cancer, Cell morphology, Cellular development

Genes in bold and italic – over-expressed, small font – suppressed.

*- genes appearing in more than in one network.

The gene ontology analysis using the IPA tool showed that many functions were identified as high-level functions (Table 3). Fischer's exact test was used to calculate a p-value determining the probability that each biological function and/or disease assigned to that data set is due to chance alone. Of these, twenty genes were revealed as involved in cancer process (*P *= 7.05E-5 to 2.92E-2).

**Table 3:** Functional Analysis*

**High Level Function**	**Significance**	**# Associated Genes**
Cell Death	1.10E-6 – 2.92E-2	22
Cell Cycle	8.85E-6 – 2.44E-2	17
Hematological Disease	2.29E-5 – 2.92E-2	15
Immunological Disease	2.57E-5 – 2.92E-2	9
Inflammatory Disease	3.55E-5 – 1.96E-2	9
Cancer	7.05E-5 – 2.92E-2	20
Cellular development	7.05E-5 – 2.92E-2	15
DNA replication, and repair	7.10E-5 – 2.92E-2	8

*Identified the biological functions and/or diseases that were most significant to the data set.

## Discussion

Tumorigenesis is a multistep process characterized by a myriad of genetic and epigenetic alterations. The evolution of human cells is driven by the aberrant function of genes that positively and negatively regulate various aspects of the cancer phenotype, including altered responses to mitogenic and cytostatic signals, resistance to programmed cell death, immortalization, neo-angiogenesis, and invasion and metastasis. Therefore identification of individuals at greatest risk of developing lung cancer at the initial steps of cancer development could enhance the efficacy of intervention modalities, thereby greatly reducing mortality from this disease.

We compared gene expression profiles of non-malignant lung tissue from patients with AC, smokers and non- smokers, matched for clinical staging all were at stage 1 besides 1 from each group at stage 2 and histologically had well differentiated adenocarcinoma. One can argue why not using matched smokers as control to those who developed already lung cancer? Both have the same risk factor to develop lung cancer but this doesn't preclude future lung cancer in the control group. Therefore they weren't useful for detection of markers for developing lung cancer in smokers.

Thirty six genes were differently expressed in smokers non-cancerous lung tissue compared to same tissue of non-smokers. Many of these genes are involved in the cancer process as described above. Smoking is the major risk factor for developing lung cancer but also it is a major risk factor for COPD, which is a diffuse pulmonary inflammatory and obstructive lung disease. Furthermore, it is an established fact that airflow obstruction, measured by simple spirometry, is an independent risk factor for the development of lung cancer. Some studies [15,16] have reported an increased risk of four to six times that of patients without airflow obstruction. The exact mechanism of the impact of COPD on the development of lung cancer is unknown [17]. A better genes isolation by addition of control groups from smokers only or COPD patients lung may be hampered by the fact that they may contain cells in precancerous or even cancerous changes, and various degree of inflammation. We also initially examined 4 patients with metastatic colon adenocarcinoma. Since their gene expression was different from lung adenocarcinoma as expected, it was stopped due to lack of relevance.

This study was based on the observation that in smokers, even the non-cancerous tissue demonstrates widespread morphological changes and tumors may arise within the genetically abnormal cells [18]. In contrast, adenocarcinoma in nonsmokers more likely arises in a field of relatively normal cells, as might be the case with prior infection, such as seen in "scar carcinomas."

As mentioned smoking induce changes of variable magnitude to the airways epithelia. Some of these subjects develop COPD, some develop lung cancer, and some both. RNA gene expression might be changed by all the described above. In addition, the presence of tumor itself may induce some inflammatory changes i.e. genes changes. The variety of genes obtained represent specific changes occurring in lung of smokers who develop AC of lung. It should be emphasized that the so called field cancerization may not always develop lung cancer and therefore these changes are not a one way road. Since the inflammatory and cancer development are a dynamic processes the genes in the network may change their state of expression, to be precise their activity. A biological progression model can be postulated in which field development plays a central role and its network gene expression change reciprocally. Consequently, monitoring of field may have profound implications for cancer detection and prevention. In view of the fact that detection of lung cancer in early state provide a better prognosis it's One of the major objective to early detect lung cancer is sampling of the suspected material. Usually the most convenient materials are those originated from blood or sputum because of simple to obtain. The value of genes expression changes and their location in biological networks, may serve as a warning for smokers and ex-smokers for the higher risk of developing lung cancer, i.e. early detection of lung cancer. Since these changes are as described field distributed the sampling could be regional rather than focal. Therefore, sputum sample cells may represent cells within the "field" and altered with genetic precancerous changes.

## Conclusion

We described 36 genes that may represent the responsible genes for enhanced risk for developing lung cancer in smokers. The relatively small sample size may contribute to reduced power of the study results. Movement towards the development of consortia to pool findings across studies and increase sample size and power will address some of this issue.

## Competing interests

The authors declare that they have no competing interests.

## Authors' contributions

DS plan the study made all coordination and was involved in the laboratory processing. IB carried out all the surgical procedure and staging and handling the samples. JS responsible for tissue diagnosis and preparing samples for RNA testing. All authors read and approved the final version of manuscript.
